# A novel CT-based automated analysis method provides comparable results with MRI in measuring brain atrophy and white matter lesions

**DOI:** 10.1007/s00234-021-02761-4

**Published:** 2021-08-14

**Authors:** Aku L Kaipainen, Johanna Pitkänen, Fanni Haapalinna, Olli Jääskeläinen, Hanna Jokinen, Susanna Melkas, Timo Erkinjuntti, Ritva Vanninen, Anne M Koivisto, Jyrki Lötjönen, Juha Koikkalainen, Sanna-Kaisa Herukka, Valtteri Julkunen

**Affiliations:** 1 University of Eastern Finland, Institute of Clinical Medicine / Neurology, P.O. Box 1627, (Yliopistonranta 1 C), 70211 Kuopio, Finland; 2 Neurosurgery of NeuroCenter, Kuopio University Hospital, P.O. Box 100, 70029 KYS Kuopio, Finland; 3 Department of Neurology, University of Helsinki and Helsinki University Hospital, P.O. Box 340, 00029 HUS Helsinki, Finland; 4 Division of Neuropsychology, HUS Neurocenter, Helsinki University Hospital and University of Helsinki, Helsinki, Finland; 5 Department of Psychology and Logopedics, Faculty of Medicine, University of Helsinki, Helsinki, Finland; 6 Department of Radiology, Kuopio University Hospital, P.O. Box 1777, 70211 Kuopio, Finland; 7 University of Eastern Finland, Institute of Clinical Medicine / Radiology, P.O. Box 1627, (Yliopistonranta 1 C), 70211 Kuopio, Finland; 8 Department of Neurology, Kuopio University Hospital, P.O. Box 1777, 70211 Kuopio, Finland; 9 Department of Neurosciences, Department of Geriatrics / Rehabilitation and Internal Medicine, University of Helsinki, Helsinki University Hospital, P.O. Box 340, 00029 HUS Helsinki, Finland; 10 Combinostics Oy, Hatanpään valtatie 24, 33100 Tampere, Finland

**Keywords:** Alzheimer’s disease, Atrophy, Computed tomography, Computer-assisted image analysis, Magnetic resonance imaging, Neurodegenerative disease

## Abstract

**Purpose:**

Automated analysis of neuroimaging data is commonly based on magnetic resonance imaging (MRI), but sometimes the availability is limited or a patient might have contradictions to MRI. Therefore, automated analyses of computed tomography (CT) images would be beneficial.

**Methods:**

We developed an automated method to evaluate medial temporal lobe atrophy (MTA), global cortical atrophy (GCA), and the severity of white matter lesions (WMLs) from a CT scan and compared the results to those obtained from MRI in a cohort of 214 subjects gathered from Kuopio and Helsinki University Hospital registers from 2005 - 2016.

**Results:**

The correlation coefficients of computational measures between CT and MRI were 0.9 (MTA), 0.82 (GCA), and 0.86 (Fazekas). CT-based measures were identical to MRI-based measures in 60% (MTA), 62% (GCA) and 60% (Fazekas) of cases when the measures were rounded to the nearest full grade variable. However, the difference in measures was 1 or less in 97–98% of cases. Similar results were obtained for cortical atrophy ratings, especially in the frontal and temporal lobes, when assessing the brain lobes separately. Bland–Altman plots and weighted kappa values demonstrated high agreement regarding measures based on CT and MRI.

**Conclusions:**

MTA, GCA, and Fazekas grades can also be assessed reliably from a CT scan with our method. Even though the measures obtained with the different imaging modalities were not identical in a relatively extensive cohort, the differences were minor. This expands the possibility of using this automated analysis method when MRI is inaccessible or contraindicated.

**Supplementary Information:**

The online version contains supplementary material available at 10.1007/s00234-021-02761-4.

## Introduction

Throughout its existence, brain imaging has been a key element in the diagnostic workup of neurodegenerative diseases. Until the 21^st ^century, the main purpose of neuroimaging was to rule out possibly treatable or causative lesions of cognitive symptoms, such as tumors, hematomas, or hydrocephalus [1, 2]. However, a growing body of evidence demonstrating the power of biological and imaging biomarkers has led to a shift in the diagnostic setup from simply excluding other diseases to also actively detecting pathological changes due to neurodegenerative diseases, e.g., Alzheimer’s disease (AD) [3–5].

According to the European Federation of Neurological Societies (EFNS) guidelines [6] regarding the diagnosis of neurodegenerative disorders, brain atrophy can be evaluated with visual rating scales in temporal areas (medial temporal lobe atrophy, MTA) [7], posterior areas (posterior cortical atrophy) [8], and globally in the whole brain (global cortical atrophy, GCA) [9], while white matter lesions (WMLs) relating to vascular pathologies are usually evaluated by the scale developed for magnetic resonance imaging (MRI) by Fazekas et al. [10]. These kinds of visual rating scales are fast and straightforward to use in clinical practice. However, they require the expertise of a neuroradiologist and are still relatively coarse, subjective and might be prone to floor and ceiling effects [11] and dependent on the experience of the image reader. These issues have been shown to cause significant intra- and interrater variability in the results [7]. The quantification of brain structures based on manual delineation is considered the ground truth, but it is very time-consuming and still partly subjective regardless of the application of carefully planned procedures [12, 13].

Recent progress in computer science and the application of machine learning methods to analyze imaging data has allowed the development of fully automated structural image analysis methods and diagnostic decision support algorithms [14]. Compared to visual assessment, automated methods provide several advantages: i) they do not require manual work and are thus user-friendly; ii) they are objective and provide reproducible results; and iii) they provide single-subject level quantitative data on brain structures that can be easily used in further analyses and computational diagnostic tools, such as the disease state index [15]. Sophisticated methods can be used to measure anything from a single brain structure to all cortical and subcortical regions in the whole cerebrum simultaneously [16–23].

To date, automated methods have focused mainly on MRI, thus excluding patients for whom only computed tomography (CT) is available. MRI is reasonably widely available and noninvasive in terms of ionizing radiation and provides precise structural information on the central nervous system. However, there are several situations in which CT might be chosen over MRI. First, some patients have contraindications to MRI, such as certain types of pacemakers. Second, a patient might be unwilling or unable to undergo the time-consuming MRI procedure because of claustrophobia or cognitive problems, causing a lack of sufficient cooperation, or just because of the prolonged duration in the supine position. Third, cognitive disorders are sometimes diagnosed in clinics without access to MRI, or a lack of resources limits the usage of MRI in these patients. Fourth, brain CT is commonly performed as part of the diagnostic procedure for various acute neurological medical conditions. These images are useful and important if a patient later develops symptoms of neurodegenerative disease or if a longitudinal assessment of structural brain changes is needed as a part of disease state follow-up.

In this study, we aimed to overcome these limitations by developing a novel automated method providing single-subject level information on structural brain changes as well as WMLs from CT images simultaneously. We compared the results from our automated analysis pipeline to those obtained from an automated MRI analysis in a multicenter cohort of 214 subjects collected from the registries of the Kuopio and Helsinki University hospitals.

## Materials and methods

### Study subjects

This multicenter study was conducted by collecting data retrospectively from the biomarker register of the University of Eastern Finland (UEF) and the Helsinki University Hospital (HUS) clinical image archive. Since the objective of the study was to compare measures obtained by CT and MRI, all subjects who were not scanned with both imaging modalities were excluded. The same exclusion criteria were applied to both UEF and HUS data. All subjects with major focal pathologies, such as hematomas (except microbleeds), demyelinating lesions, cortical infarcts, traumatic brain lesions, or tumors, were excluded. Subjects with minor focal pathologies, such as small lacunar or cerebellar infarctions, were not excluded.

All subjects from the UEF biomarker register were assessed and imaged at Kuopio University Hospital (KUH) between 2004 and 2017 and were referred to the participating outpatient clinic due to suspected cognitive decline; these patients were examined in accordance with the national Finnish guidelines for the diagnosis of neurodegenerative diseases [24]. For the UEF data, the time interval between the CT and MRI scans was required to be less than 6 months to avoid differences in brain structures due to possible disease progression. In most cases, the reason for initial brain imaging was to exclude focal pathologies as the cause of cognitive or neurological symptoms. If initial imaging was performed using CT, the usual rationale for subsequent MRI was to obtain more precise information on brain structures or possible pathological changes for differential diagnostic purposes. In some cases, CT was performed after MRI, mainly because of new acute neurological symptoms, such as disorientation or vertigo.

Image data from the Helsinki area were collected from the HUS clinical image archive from January 2014 to December 2016. The HUS clinical image archive contains images from HUS and from five area hospitals in the Helsinki region. The brain images were systemically screened by qualified healthcare professionals to make CT-MRI image pairs. For all cases in the HUS cohort, the time between scans was less than 6 weeks. In conclusion 147 CT-MRI image pairs were divided into three Fazekas groups (Fazekas 0–1, *n* = 50; Fazekas 2, *n* = 48) [25].

### Image acquisition

Since imaging was performed at multiple sites and the data were collected over a long period of time between 2004 and 2017, the data contained images obtained using several different scanners from various manufacturers. All MRI scans were performed using either a 1.5T or 3T MRI scanner manufactured by Siemens or Philips. Automated MRI segmentation and structural analysis were performed on T1-weighted images with a three-dimensional magnetization-prepared rapid acquisition gradient-echo (3D-MPRAGE) sequence or a corresponding sequence. Other imaging parameters varied slightly, but all T1-weighted images had a slice thickness of 0.9 – 1.5 mm, a voxel volume of 0.2 – 1.6 mm^3^, and full coverage of the skull and brain. WMLs were segmented from axial images obtained with a fluid-attenuated inversion recovery (FLAIR) sequence with varying slice thicknesses of 0.6 – 6.5 mm and voxel volumes of 0.1 – 5.3 mm^3^. CT images were acquired with Siemens and GE devices with a slice thickness of 1.0 – 5.5 mm, a voxel volume of 0.1 – 1.4 mm^3^, and an orientation aligned along the skull base.

**Fig. 1:**
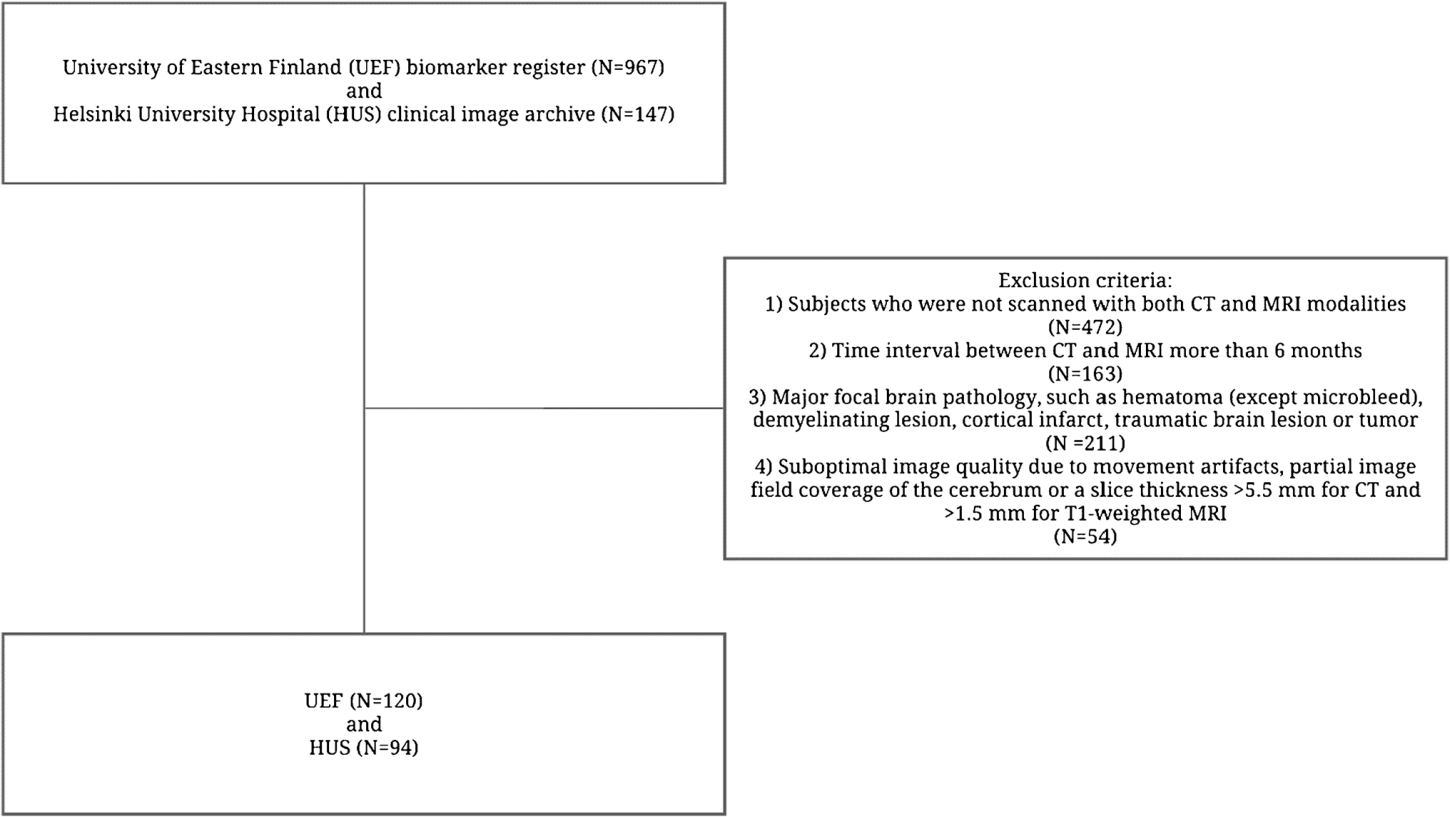
Flowchart describing the procedure for study subject selection

Finally, subjects with suboptimal image quality, e.g., due to movement artifacts, partial image field coverage of the cerebrum, or a slice thickness >5.5 mm for CT and >1.5 mm for T1-weighted MRI, were excluded. This led to the inclusion of 214 subjects, 120 and 94 from the UEF, and HUS databases, respectively, with a mean age of 69.9 ± 9.8 years in total. A flowchart describing the subject selection protocol is presented in Fig. 1.

### Processing of MRI images

The MRI images were analyzed using the cNeuro quantification tool (Combinostics, Ltd.). The tool segmented the 3D T1-weighted images into 133 structures using a multiatlas segmentation method. cNeuro tool also segmented WMLs on FLAIR images. The MTA, GCA, and Fazekas grade were determined from these results using the method described in [26]. In short, first a linear regression model was used to estimate the visual grade from the automatically determined measures. Thus the estimate was fine-tuned using a piece-wise linear regression model. The regression parameters were obtained from a separate training set (n = 513) with visual MTA, GCA and Fazekas grades available. The MTA values (left and right, continuous values between 0 and 4) were determined from the volumes of the inferolateral ventricles and hippocampus, the GCA was computed from the VBM results, and the Fazekas grade was determined from the volume of WMLs in deep white matter [26].

### Processing of CT images

**Fig. 2:**
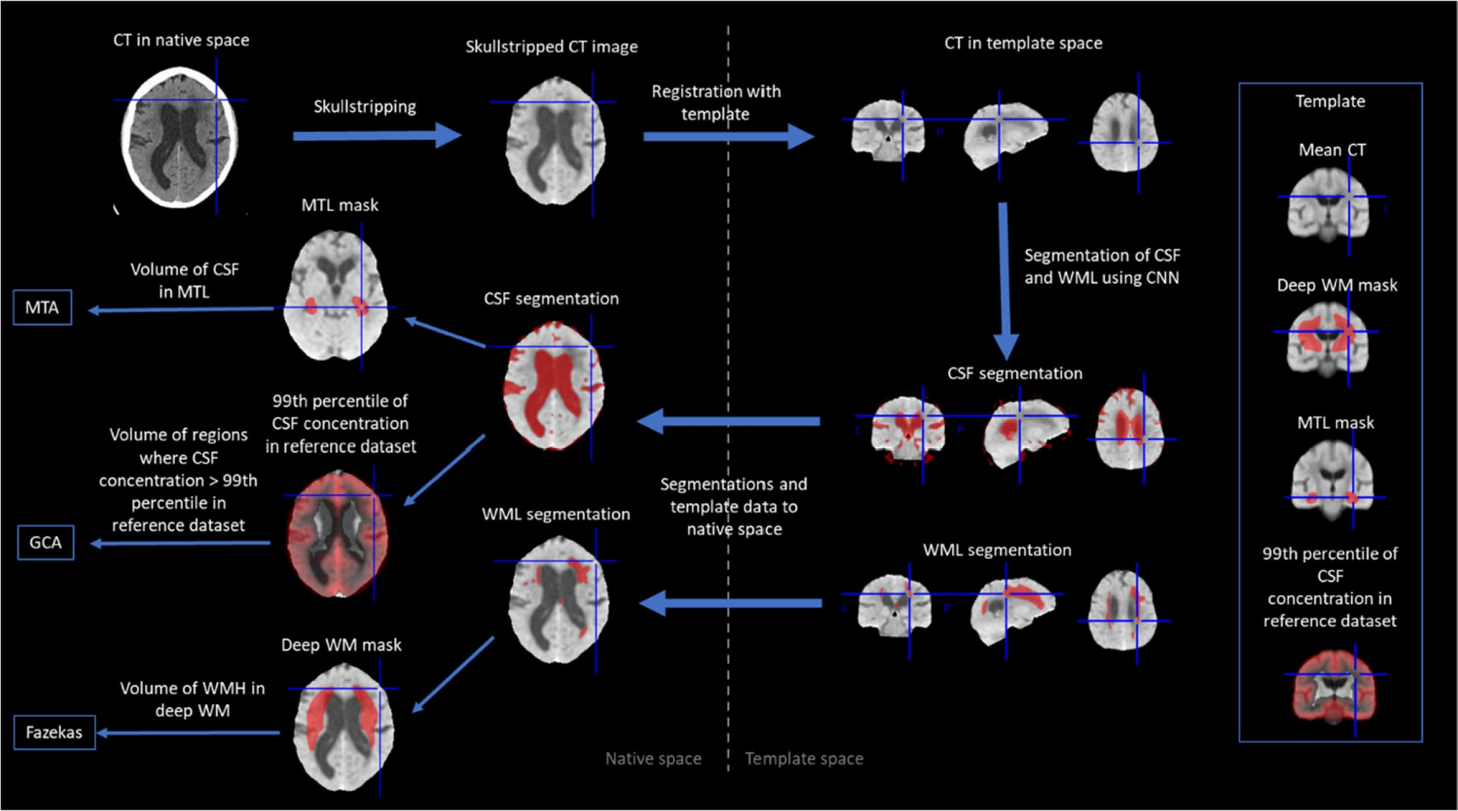
Flowchart of CT image analysis procedure

The analysis of CT images is summarized in Fig. 2. First, skull stripping was performed on the CT image. Then, the image was registered with a mean anatomical CT template. Convolutional neural network (CNN) segmentation was performed in this template space to segment cerebrospinal fluid (CSF) and WMLs. The segmentation results and predefined data in the template space were then transformed back to the native space of the CT image. Finally, features were computed from the segmentation results, and the computational counterparts for the MTA, GCA, and Fazekas grades were determined for the CT image.

### Template data

The template data consist of a mean anatomical CT template representing both the mean anatomy and mean intensity of CT images. The CT template was generated by first rigidly registering the CT images of the dataset with the corresponding T1-weighted images. Then, the T1-weighted images were registered with the mean anatomical template of cNeuro, and the CT images were transformed using the same transformations. Finally, the mean intensities of CT images were computed.

In addition, masks defining the deep white matter (for computation of the Fazekas grade) and a mask for the medial temporal lobe (MTL) were manually drawn from the template. Furthermore, a probabilistic map of the CSF concentration in cognitively normal subjects was computed from a multicenter dataset of 835 subjects by registering the MRI images of the subjects to the mean template, computing the CSF concentration in each voxel and defining the 99^th ^percentile of the CSF concentration. It was assumed that the voxels where the CSF concentration was higher than the 99^th ^percentile of the reference dataset represented abnormal CSF, i.e., brain atrophy, and these voxels were consequently used to compute the GCA grade.

### Skull stripping

As the first step, the brain was extracted from the CT images using the expectation–maximization (EM) algorithm. The steps are summarized below:

1. Filter image using nonlocal means smoothing.
2. Remove outlier intensities.
3. Two-class EM classification using fixed priors.
4. Compute threshold for the skull from the EM results.
5. Morphological operations to fine tune the brain mask.

### Registration with mean template

The target CT image was registered with the mean CT template using affine registration. The 9-parameter affine registration was performed on the binary skull masks by minimizing the intensity differences using a gradient-based optimization.

The CNN computations were performed for the images in the mean CT template space. For the CNN, the intensity of the CT image was normalized by z-scoring the brain voxels.

To accurately transform the template data (deep WM mask, MTL mask, and CSF percentile data) to the subject CT image, the result of the affine registration was refined by the nonrigid registration of the subject CT image and the mean CT template using maximization of the normalized mutual information of the grayscale images.

### CNN segmentation

The CNN was used to segment the CSF and WMLs from the CT images. To establish the ground truth segmentations for training, the CSF and WMLs were segmented from the MRI images using cNeuro. Then, the segmentations were propagated to the subject CT space using rigid T1-CT registration and finally to the mean CT template space using the affine registration described above.

In this work, we used a U-shaped CNN [27, 28]. CNNs are machine learning models that take a large number of training samples as an input and build a model that will predict the output based on the training samples. The CNN architecture used in this work was the U-shaped residual network presented by Guerrero et al. [27]. In short, the network consisted of 12 layers with approximately one million parameters. There were 8 residual elements, 3 deconvolutional layers, and a final convolutional layer that provided the class probabilities for each voxel as an output. The training of the CNN model was performed using tenfold cross-validation, i.e., 90% of the dataset was used in training and the remaining 10% in testing. This was repeated 10 times so that each image was once used in the test set.

In addition to the cross-validation process, CNN segmentation was repeated ten times such that ten separate segmentations were obtained for each CT image. The objective was to improve the robustness by combining the ten segmentations. The combination of the ten segmentations was performed as follows:

1. Compute the correlation coefficient between each segmentation pair, *c*_*ij*_ (10×10 matrix of correlation coefficients). The output of the CNN is the probability of the object.
2. For each segmentation *i*, compute the number of *c*_*ij*_ > min(0.8, 0.9*max(*c*_*ij*_)), *n*_*i*_.
3. Compute the weight *w*_*i*_ = *n*_i_ / sum(*n*_i_).
4. Compute the weighted sum of the original segmentation probabilities, and threshold the result with the threshold 0.5.

The combination of the segmentations was performed in the template space. Thereafter, the final segmentation using the affine transformation was computed in the preprocessing phase. The MTL and deep WM masks and the CSF percentile data were propagated to the native CT space using the affine and nonrigid transformations. All the remaining computations were performed using the data in the native CT space.

### Computational measures from CT measures

The method to define the computational measures from one or many imaging measures has been previous described in detail [26] but is briefly summarized here. The method is based on a training set, where the ground truth grade is available. In [26], the ground truths were visual grades, but in this study, we used the computational MRI grades as ground truths. First, a linear regression model was trained to estimate the grades from the CT measures. Then, a piecewise linear model was used to match the median values of the ground truth grades and estimated grades. This two-step model was then applied to unseen data to define the computational measures from CT measures.

### Computational MTA grade from CT

The CT estimate of MTA was computed from the volume of CSF within the MTL mask. The CSF volume was normalized to the total brain volume (computed as the volume of the skull-stripped CT image).

### Computational GCA grade from CT

To determine the computational GCA, the CSF probability (CSF segmentation without the final thresholding) of the CT image was compared to the 99^th^ percentile of the reference dataset of CSF probabilities. The volume of the regions where the CSF probability of the CT image was higher than the 99^th^ percentile was computed. The volume normalized for the total brain volume was used as the measure for GCA.

**Fig. 3:**
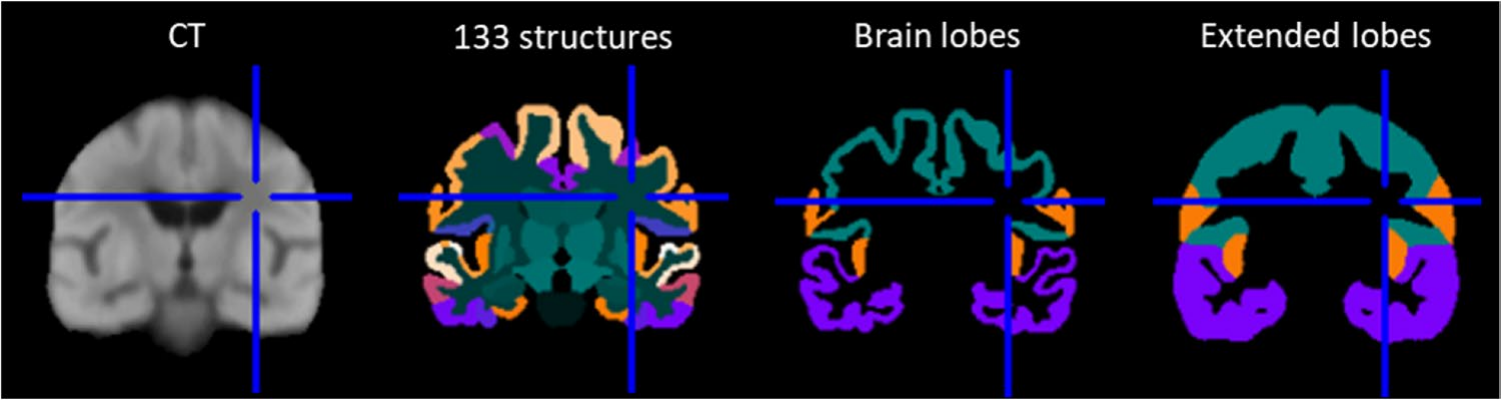
Generation of template masks for the computation of GCA for each brain lobe. The segmentations of the lobes were first obtained by combining original cortical regions. Then, the segmentation was extended to CSF outside the brain by labeling each CSF voxel to the closest lobe

The GCA grades were also computed for each brain lobe (frontal, temporal, parietal, and occipital lobes). The computation was identical to the global GCA, but the CSF volumes were computed only for one lobe at time. The segmentation of the lobes was obtained by transforming the template segmentation (Fig. 3) to the subject CT image using the same transformations used for other template data (e.g., MTL mask; see Fig. 2).

### Computational Fazekas grade from CT

For the estimation of the Fazekas grade from CT images, the volume of WMLs inside the deep WM mask was computed and normalized to the total brain volume.

### Evaluation and statistical methods

The CT-based measures of MTA, GCA, and WMLs were compared to corresponding computational measures from MRI. Although the original Fazekas grade [10] refers solely to an MRI-based evaluation, in this study, we describe the CT-based WML measures with the same scale to avoid unnecessary complexity. We computed the Pearson correlation coefficient for these measures and the number of cases where the estimates were identical for CT and MRI. We also assessed the proportion of cases where the difference in the estimated atrophy or Fazekas grade was at most one. When calculating the correlation coefficient, we used continuous values for MTA/GCA/Fazekas given by the image analysis pipelines. The percentages of estimation errors of the CT- and MRI-based measures were based on categorical class numbers. The categorical classes were obtained from the calculated continuous values by first cutting the value to the allowed range ([0 3] or [0 4]), and then the value was rounded to the nearest integer (0, 1, 2, 3, or 4 for MTA; 0, 1, 2, or 3 for GCA and Fazekas). The agreement between CT- and MRI-based values was also assessed by calculating Cohen’s quadratic weighted kappa for each result. The results were computed using MATLAB R2016a, MathWorks, Inc., and the CT and MRI measures were visualized using Bland–Altman plots and scatter plots.

### Statement of ethics

The usage of UEF biomarker register data was approved by the Research Ethics Committee of the Northern Savo Hospital District, Kuopio, Finland. HUS did not require additional review by the ethical board for the retrospective analysis of imaging data collected prospectively as part of routine clinical care at the time the study was done. The study was conducted in accordance with the principles of the Declaration of Helsinki and had institutional approval from each participating center. All imaging data were anonymized and handled with discretion.

## Results

The Pearson correlation coefficients, the numbers of identically estimated grades, estimation error rates, and Cohen’s kappa values for intermodality agreement for the computational CT and MRI measures are presented in Table 1. From the clinical perspective, it is important to distinguish whether there is none or only minor changes (corresponding to grades 0–1) or clearly noticeable changes (grades over 1). Therefore, we did an analysis with these combined groups of normal or only minor changes versus abnormal grades. The results are displayed in Table 1 in the % of identical normality classification column.

**Table 1:** Correlation coefficients and percentages describing differences between estimated grades and quadratically weighted Cohen’s kappa values for the computational MTA, GCA, and Fazekas grades between CT- and MRI-based measures

	Correlation (r)	% of identical estimated grades	% estimate error of grades ≤ 1	% of identical normality classification	Quadratically weighted kappa
MTA, right	0.91	58	98	86	0.83
MTA, left	0.89	60	98	90	0.86
MTA	0.90	60	97	90	0.84
GCA	0.82	62	98	84	0.78
Fazekas	0.86	60	98	88	0.82

**Fig. 4:**
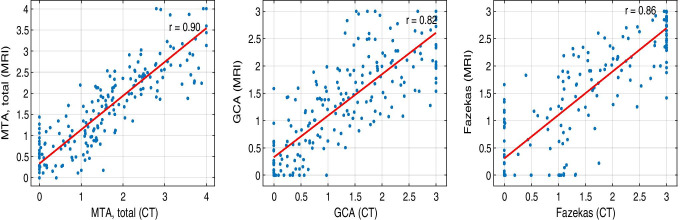
Scatter plots demonstrating the correlation of the computational MTA, GCA, and Fazekas grades estimated from MRI and CT

**Fig. 5:**
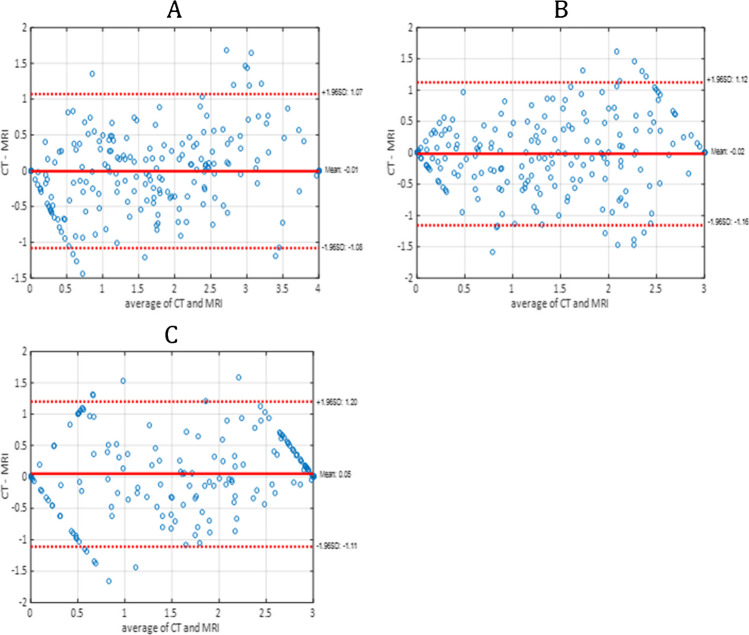
Bland–Altman plots for the **A** MTA, **B** GCA, and **C** Fazekas grades from CT and MRI

Table 2 shows the confusion matrix for the class distribution for the MTA, GCA, and Fazekas values of the study cases. Scatter plots and Bland–Altman plots for these measures are presented in Figs. 4 and 5, respectively. For the Pearson correlation coefficients and percentages describing differences between estimated grades regarding GCA in different cerebral lobes, see Table 3.

**Table 2:**
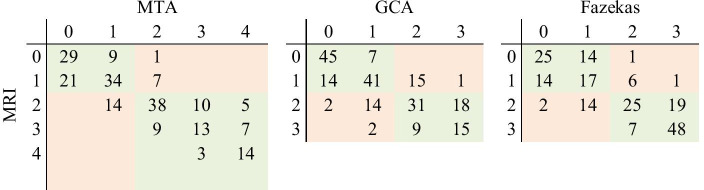
Confusion matrices for the computational MTA, GCA and Fazekas grades from MRI and CT. Green color indicates the number of correctly classified normal/abnormal subjects, and red color shows the number of incorrectly classified subjects

**Table 3:** Correlation coefficients and percentage of correctly estimated grades for the computational GCA for each lobe between CT- and MRI-based values

	Correlation (r)	% of identical grades	% estimate error of grades ≤ 1
Frontal	0.81	56	97
Temporal	0.86	63	98
Parietal	0.75	61	93
Occipital	0.71	56	86

## MTA

MTA had the highest correlation between the two imaging modalities, at r = 0.90 (Table 1, Fig. 4). The percentage of identically estimated MTA measures was 60%. In 98% of the cases, the difference in the MTA value between the CT- and MRI-based measures was equal to or less than one. Quadratically weighted Cohen’s kappa values for MTA showed excellent agreement between the two modalities (Table 2). According to the Bland–Altman plot (Fig. 5), CT tended to slightly underestimate atrophy grades at lower values and overestimate those in the severely atrophied brain compared to MRI. With combined groups of normal or only minor changes versus abnormal grades, the accuracy was up to 89.7%.

## GCA

GCA grades between CT and MRI had a high correlation coefficient (r = 0.82) (Table 1, Fig. 4). In 96% of the cases, the difference in the GCA value was equal to or less than 1 (Table 1). Weighted kappa values presented excellent agreement between CT and MRI (quadratic kappa = 0.78). The Bland–Altman plot displays a slight tendency of pronounced spreading for the higher GCA values (Fig. 5). However, neither modality seemed to present any systematic differences in the computed values. The accuracy for the combined groups normal versus abnormal classification was 84%.

The results for cortical atrophy in each lobe separately are shown in Table 3. CT and MRI seemed to provide similar results, especially in the frontal and temporal lobes, whereas the correlation was slightly weaker in the parietal and occipital lobes. However, estimation differences between the whole atrophy grades were rare in the frontal, parietal, and temporal lobes (93–98% of results are within the same grade). Even in the weakest area, that is, the occipital lobe, 86% of the cases were within the range of 1 atrophy grade from each other.

### Fazekas

The Fazekas grades presented a high correlation coefficient of r = 0.86 (Table 1, Fig. 4). The grades obtained by CT and MRI were identical in 60% of the cases, but the difference between the Fazekas grades was at most one in 97% of all cases (Table 1). The weighted kappa values also showed excellent agreement between the values obtained by different modalities. The spread of values was pronounced at lower Fazekas grades, as expected (Fig. 5). For Fazekas the accuracy with combined normal and abnormal groups was 88%.

## Discussion

The aim of this study was to compare automated quantitative analysis methods measuring brain atrophy (MTA, GCA) and WMLs between CT and MRI as the gold standard in a large multicenter cohort of 214 subjects. According to our results, the measures obtained with our novel CT algorithm correlate strongly with equivalent MRI-based values and provide comparable information on atrophy rates and WMLs in most cases. Although the estimated atrophy and Fazekas grades did not have a particularly high rate of *exact* agreement between the two modalities, the errors in estimated grades were only minor, as the difference was equal to or less than one in 86–98% of cases. The CT-based measures had a tendency for slightly higher MTA and GCA grades than the MRI-based measures in subjects with severe brain atrophy. In well-preserved brain parenchyma, the effect was opposite in the MTL region. When assessing the brain lobes separately, cortical areas in the parietal and occipital lobes demonstrated slightly lower correlations between the CT and MRI measures than the frontal and temporal regions. It should be noted, however, that severe errors of 2 or more grades were also rare in the parietal and occipital regions. The detection of WMLs according to the Fazekas scale showed a high overall correlation between the two imaging modalities, although the Bland–Altman plot (Fig. 5) shows that among the cases with only minor WMLs (Fazekas grades 0–1), there is heavy scattering in the estimated values. This indicates that the detection of WMLs with CT is likely more reliable in cases with pronounced WMLs. We also analyzed correlations of the original MRI and CT features that were used to generate the MTA, GCA and Fazekas grades, which are presented in supplementary Figures S1, S2, S3, S4, and S5. The correlations of the MRI and CT features were equivalent with the transformed MTA, GCA, and Fazekas grades correlations.

Earlier studies have shown that radiologists can visually assess brain atrophy using both imaging modalities with almost equal accuracy [29, 30]. The comparison of atrophy detection between CT and MRI using visual rating scales was later replicated with more advanced 64-detector row CT, with similar results [31]. MRI was found to be more sensitive in showing signs of WMLs, but this has not been considered a remarkable issue, as minor WMLs do not cause clinically relevant symptoms, whereas major WMLs indicating a vascular etiology as the probable reason for cognitive decline are also detected on CT [31].

Some studies have also applied automated image analysis methods to CT images. Chen et al. [32] assessed WML volumes on CT images automatically by applying a random forest method to a large dataset of 1082 acute ischemic stroke patients. They showed that the CT-based WML volumes had a high correlation with the results obtained using MRI. Similar results were reported in two recent publications, particularly in patients with a moderate or severe WML load [25, 33]. However, these studies focused solely on WMLs, providing no information on structural features of the brain that are important in the differential diagnosis of neurodegenerative diseases. This issue was addressed by Imabayashi et al. [34], who developed a voxel-based morphometry (VBM)-based technique to detect brain atrophy on CT images. Their results demonstrate statistically significant differences between groups of controls and AD patients. However, the population in this study was very small, and the method focused on detecting groupwise differences rather than measuring atrophy at the single-subject level.

Our results are in line with those of earlier studies comparing neuroimaging by CT and MRI. Wattjes et al. [31] showed that MTA and GCA can be assessed visually from brain CT and MRI with excellent intraobserver agreement. In our previous work, the computational MTA, GCA, and WML grades from MR images showed mainly high correlation with visual grades; MTA-left (training set 0.83/test set 0.78), for MTA-right (training set 0.83/test set 0.79), and for WML (training set 0.75/test set 0.75), except for GCA (training set 0.64/test set 0.64) [26]. Computational WML grades were equal to visual grades in 78% of cases from both CT and MR images [25]. In this study, the results regarding the Fazekas grade were slightly weaker, but there was still substantial overall agreement. Most of the discordant Fazekas grades between CT and MRI dealt with lower grades of 0 and 1, a finding that can also be seen in our results (Fig. 5). This phenomenon is likely caused by the better sensitivity of MRI in detecting minor WMLs, which has been reported earlier in several studies [29–31]. Studies utilizing *automated* CT methods in analyzing WMLs have also shown a strong correlation with the Fazekas grade evaluated visually on MRI by trained experts, especially in the higher Fazekas grades of 2–3 [25, 32, 33]. In this study, we particularly compared the correlation between computational CT and MRI grades, which excludes inter- and intrarater variations.

To date, there have been only a few studies concerning automated structural analysis of the brain based on CT images. Adduru el al. [35] developed a method called “CTseg” to automatically measure the total intracranial volume and total brain volume from a CT scan. Their results showed excellent correlation with automatically and manually estimated volumes. However, these measures are quite coarse, as they do not provide any information on the distribution of possible atrophy, which is particularly interesting in the differential diagnosis of neurodegenerative diseases, such as AD and frontotemporal dementia (FTD). Imabayashi et al. [34] compared atrophy rates groupwise between 7 controls and 5 AD patients measured by the automated VBM method using CT and MRI scans of the same study subjects. CT-based evaluation showed significantly atrophied areas in the hippocampal region in the AD group, as did MRI. Surprisingly, CT also seemed to detect significant atrophy in the temporopolar areas, caudate nucleus, and anterior cingulum, where MRI did not. The authors concluded that CT-based analysis might be even more sensitive to brain atrophy than MRI, possibly because of the greater homogeneity and lesser distortion of CT images than MRI images. However, their study population was very small, which sets limitations on the reliability and generalizability of the results. Based on our results, the atrophy measures obtained from CT showed excellent agreement with those obtained from MRI in general but might present either slight over- or underestimation of the structural volumes depending on the grade of brain atrophy (Fig. 5). In small sample sizes, this effect could significantly contribute to the results.

Our study has several strengths and advantages. The study population is larger than that of other neuroimaging studies with similar goals and methods. The usage of multiple centers, scanners and imaging systems requires certain methodological robustness, and we did not encounter any major segmentation errors or other technical issues in the pipeline.

Our results are in line with those previously reported in the literature and provide high agreement between values originating from CT and MRI modalities. Similar results have been acquired previously in smaller studies mentioned above but with the limitation of concentrating only on WMLs [25, 32, 33], assessing only groupwise differences [34] or assessing very coarse structural features, such as the whole brain volume [35]. Our study provides an improvement over these methods by offering a more comprehensive analysis of the brain simultaneously.

Certain limitations of our study should also be addressed. First, one could argue that differences in the image acquisition protocols and equipment might have had an impact on the results. This is a common issue in imaging studies, with no final solution. Previously published data suggest that at least variation in the MRI scanner manufacturer, pulse sequence or spatial resolution does not have a significant effect on fine structural MRI measures, such as the cortical thickness [36]. The scanners used in clinical practice usually vary depending on the center, meaning that a setting with multiple scanners represents real-world circumstances better than a strictly planned imaging protocol. Additionally, our results are logical and consistent with those previously reported in the literature without unexplainable deviations, suggesting that our methodology is most likely quite robust and tolerates minor variance in the imaging data without having a significant impact on the results. The slice thickness of the CT images varied between 1.0 and 5.5 mm and was 4 or 5 mm in 87.9% of the cases. The correlation between the CT and MRI estimates did not appear to differ significantly depending on the slice thickness. However, it should be noted that our cohort had only few subjects with 1 mm slice thickness available meaning that we cannot fully assess the potential advantages of these thin slices in our cohort. This remains to be clarified in future studies.

Second, the time interval between CT and MRI varied from a few days to a maximum of 6 months. Among cases with the longest time interval of between 5 and 6 months (n = 9), there could be some progress in brain atrophy and the number of WMLs, meaning that the brain itself is not exactly similar at the time of the two imaging examinations. This could lead to increased variation in the structural measures and WML volume. However, the mean time difference in our study population was only 31 days, during which the development of new significant neurodegenerative changes is unlikely. Furthermore, possible differences in the calculated MTA, GCA, and Fazekas grades between the modalities caused by the progressing brain changes would most likely weaken the observed correlations, meaning that our results do not overestimate the accuracy of our methodology.

Third, although our results demonstrate high correlation between the estimated classes from different imaging modalities, the figures of exact agreement leave room for improvement. In clinical environment, it is important to first determine whether the patient has clearly noticeable structural changes or not. To simulate this we analyzed combined groups of minor or no changes (grades 0–1) versus clearly abnormal values (grades >1). This binary comparison showed that our method gives identical estimation of normal versus abnormal structures in 84 – 90% of the cases, which improves the percentage of identically estimated grades using this comparison, but naturally does not improve the accuracy of our method. However, these high numbers suggest that the minor differences between all classes are most likely not critical from the clinical point of view. The dataset consisted of 214 subjects that were used as a training set for CNN in a tenfold cross-validated study. The segmentation accuracy could probably be improved by increasing the size of the dataset, and in the best case utilize manual segmentations for the CT images.

The objective of this study was to compare the results from the CT imaging with the MRI findings. Therefore, automated analysis was used both for CT and MRI. However, the comparison of the results to radiologists’ visual ratings would provide more comprehensive information on the quality of our automated CT analysis results.

## Conclusion

The results demonstrate that important imaging features in the clinical evaluation of neurodegenerative disorders (MTA, cortical atrophy, and Fazekas grade) can also be assessed reliably from a CT scan with our automated analysis method. The imaging features match up exactly in ~60% of cases, but differentiate those cases with moderate to progressed structural changes from those with none or only minor findings with 84 – 90% agreement compared to MRI. This expands the possibilities of using these automated analysis methods in the clinical environment when MRI is inaccessible or contraindicated.

## Supplementary Information

Below is the link to the electronic supplementary material.

Supplementary file1 (DOCX 227 KB)
