# Supplementary material for: A novel CT-based automated analysis method provides comparable results with MRI in measuring brain atrophy and white matter lesions
Source: Neuroradiology. 2021 Aug 14;63(12):2035–46. doi: 10.1007/s00234-021-02761-4 (PMC8589740; doi:10.1007/s00234-021-02761-4)
Supplement: Supplementary file 1 — Supplementary file1 (DOCX 227 KB) [file 234_2021_2761_MOESM1_ESM.docx]

**SUPPLEMENTARY MATERIAL**

Figures S1-S5 show the correlation of the original MRI and CT features that were used to generate the MTA, GCA and Fazekas grades.

Figure S1. Correlation between the MRI volume of hippocampus and inferior lateral ventricles and the CT volume of CSF in medial temporal lobe for the right hemisphere. The correlation coefficients were -0.45 for hippocampus and 0.91 for inferior lateral ventricles.

Figure S2. Correlation between the MRI volume of hippocampus and inferior lateral ventricles and the CT volume of CSF in medial temporal lobe for the left hemisphere. The correlation coefficients were -0.52 for hippocampus and 0.92 for inferior lateral ventricles.

Figure S3. Correlation between the MRI volume of hippocampus and inferior lateral ventricles and the CT volume of CSF in medial temporal lobe for both hemispheres. The correlation coefficients were -0.48 for hippocampus and 0.92 for inferior lateral ventricles.

Figure S4. Correlation of the MRI percentage of abnormal low GM concentration and the CT volume of abnormal high CSF concentration. The correlation coefficient was 0.74.

Figure S5. Correlation of the MRI deep white matter WML volume and the CT deep white matter WML volume. The correlation coefficient was 0.89.
